# How many key informants are enough? Analysing the validity of the community readiness assessment

**DOI:** 10.1186/s13104-021-05497-9

**Published:** 2021-03-09

**Authors:** Saskia Muellmann, Tilman Brand, Dorothee Jürgens, Dirk Gansefort, Hajo Zeeb

**Affiliations:** 1 Department of Prevention and Evaluation, Leibniz-Institute for Prevention Research and Epidemiology – BIPS, Achterstr 30, 28359 Bremen, Germany; 2 Association for Health Promotion and Academy of Social Medicine Lower Saxony, Fenskenweg 2, 30165 Hannover, Germany; 3 Health Sciences Bremen, University of Bremen, Bremen, Germany

**Keywords:** Community-based health promotion, Assessment, Community readiness, Validity, Key informants

## Abstract

**Objective:**

Communities are important settings for health promotion and prevention. The community readiness assessment offers a structured approach to assess resources and opportunities to tackle a health problem within a community. The assessment relies on semi-structured interviews with key informants from the communities. A number of 4–6 key informant interviews are recommended in the literature. However, it is unclear whether this is sufficient to obtain a valid representation of the respective community. This study analysed whether increasing the number of key informants from 4–6 to 12–15 alters the results of the community readiness assessment.

**Results:**

A total of 55 community readiness interviews were carried out in 4 communities. Overall, the community readiness scores showed little variation after having interviewed 10 key informants in a community. However, even after completing 10 interviews in a community, key informants were still able to identify up to 6 new information items regarding community efforts for physical activity promotion among the elderly, contact and communication channel for informing or approaching the target group, or barriers to participation.

**Supplementary Information:**

The online version contains supplementary material available at 10.1186/s13104-021-05497-9.

## Introduction

Community-based interventions are important approaches to prevention and health promotion because they can reach their target population in their natural living environment [1]. Such interventions can initiate changes not only on the individual level but also on different levels of the social and built environment and thus may lead to sustainable health improvements [2]. While the term community covers a broad range of social groups that share a common identity or other characteristics [3], in this article community refers to small scale geographical areas where people live under the same political administration such as neighbourhoods, city districts or towns. Community-based approaches often rely on early involvement of local stakeholders and residents to foster participation and capacity building [4]. The community readiness assessment (CRA) offers a structured approach to assess resources and opportunities to tackle a health problem within a community [5]. It builds upon a stages-of-change approach and associates each community with a certain degree of preparedness ranging from no awareness to professionalization of efforts. The assessment relies on semi-structured interviews with key informants from the communities. A number of 4–6 key informant interviews are recommended in the community readiness (CR) literature [5, 6]. However, it is unclear whether this number suffices to obtain a valid representation of the community [7]. In qualitative research, the concept of data saturation refers to the point in data collection when no new information arises from subsequent data collection efforts [8, 9]. Studies examining the number of qualitative interviews required to reach data saturation found that 12 [10] to 17 interviews [8] appeared to be sufficient. Nevertheless, the number of interviews necessary to achieve a valid CR assessment in the field of physical activity (PA) promotion has not been investigated yet. As part of a community-based research project on PA promotion for the elderly (AEQUIPA – Ready to Change) [11], this study analysed whether increasing the number of key informants from 4–6 to 12–15 alters the results of the CRA.

## Main text

### Methods

#### Study population

Key informants (e.g., representatives from local public authorities, senior citizen advocacy groups, sports clubs) were either identified via online searches or recommended by other key informants. They were interviewed with an adapted version of the CRA targeting PA in older adults [12, 13]. The Ready to Change project entailed several rounds of CRA in the participating communities. In the first round in 2015, 4–6 key informants per community were interviewed. In the second round in 2018 (data base of this analysis), the key informants from the first round were interviewed again, but the sample was extended by 6–10 new key informants in each community.

### Data collection

The CRA was conducted between April and May 2018 in four communities in the Metropolitan region Bremen-Oldenburg, Germany. The sample comprised two semi-rural (both about 30,000 inhabitants) and two urban communities (districts of a larger city; 27,000 and 36,000 inhabitants, respectively). The communities were selected based on the fact they witnessed a marked increase in population ageing. Levels of deprivation and sociocultural diversity were higher in the urban communities compared to the semi-rural ones. The adapted CRA interview guide contained closed and open-ended questions addressing the five dimensions of the CR [5]: 1. Community efforts and knowledge of efforts, 2. Leadership, 3. Community climate, 4. Community knowledge of the issue, and 5. Resources. Interviews were conducted face-to-face or via telephone. All interviews were audiotaped and transcribed verbatim.

### Analysis

Based on the instructions of the CR manual [5], each interview was scored independently by two researchers to provide a CR score for each dimension and an overall CR score using a nine-point rating scale (scores between 1 = no awareness and 9 = professionalization). To examine whether increasing the number of interviews from initially 4–6 to 12–15 leads to a change in the CR score, the mean CR score of the initial sample was compared with the mean CR score of the newly identified key informants for each community. Mean differences were calculated with 95% confidence intervals (CI). For analyzing whether mean CR scores stabilize with increasing numbers of interviews, change in the mean CR score was calculated for each new key informant interviewed. Prior to the analysis, we defined that the mean CR score is stable if the change with every new respondent is ≤ 0.1 standard deviations (SD), taking Cohen’s classification of effect sizes as an orientation [14]. Therefore, we calculated change in the CR score as standardized mean differences (*Standardized mean difference* = *(CR mean score *_*(x respondents*+*1)*_*—CR mean score *_*(x respondents)*_*)/SD *_*(CR mean score)*_). The analysis of the stabilization of the mean CR scores was performed using the original order of conducted interviews and a random order of respondents. In addition to the analysis of the CR score, we wanted to find out the level of content-related saturation that was reached by increasing the number of interviews. To this end, we selected three open-ended questions from the CR interview guide and analysed whether an interview added new information regarding (a) community efforts for PA promotion among the elderly, (b) contact and communication channel for informing or approaching the target group, and (c) barriers to participation. A linear regression was performed to analyse the association between the number of interviews and newly identified issues of the CR.

### Results

A total of 55 respondents were interviewed. The number of interviews per community varied from 12 to 15. The majority of key informants were from sports clubs or sports facilities (40%) or worked for public authorities (36%). 15% were representatives from senior citizen advocacy groups and 9% worked for civil services in the communities. The mean age of the respondents was 59 years (standard deviation = 9.25, range: 29–85). 56% of the respondents were female and 44% were male. At some point during the recruitment process it appeared difficult to identify further key informants in some of the communities. This is the reason why it was not possible to obtain 15 interviews in all communities.

A comparison of the CR scores from the initial sample with the extended sample revealed mean differences of −0.45 to 0.67 CR scale points across the communities (Table 1). None of the mean differences was statistically significant.

**Table 1:** Comparison of CR score in the standard and the extended sample by community

Community	Community readiness score			Mean differences (extended–initial) (95% CI)
	Initial sample	Extended sample	Combined	
Community A	4.51 (0.72; n = 5)	5.18 (0.82; n = 10)	4.96 (0.83; n = 15)	0.67 (−0.27–1.61)
Community B	4.96 (0.66; n = 5)	4.72 (0.60; n = 9)	4.81 (0.61; n = 14)	−0.24 (−1.00–0.51)
Community C	4.97 (0.62; n = 5)	4.52 (0.66; n = 7)	4.71 (0.65; n = 12)	−0.45 (−1.29–0.38)
Community D	4.92 (0.51; n = 5)	4.69 (0.47; n = 9)	4.78 (0.48; n = 14)	−0.23 (−0.84–0.37)

Numbers are means with standard deviations in brackets

**Fig. 1:**
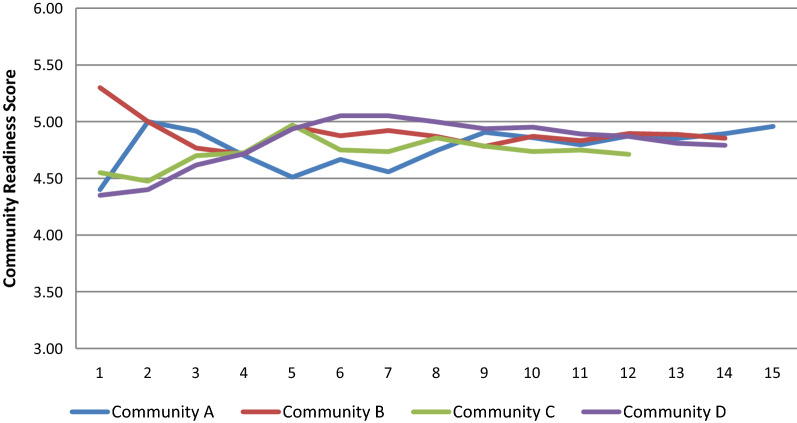
Mean CR score in 4 communities. Number of respondents are shown on the X axis

Figure 1 shows how the CR score changed with increasing number of interviews. Overall, the figure indicates a convergence of the CR scores with only little variation after having interviewed 9 or 10 key informants.

**Fig. 2:**
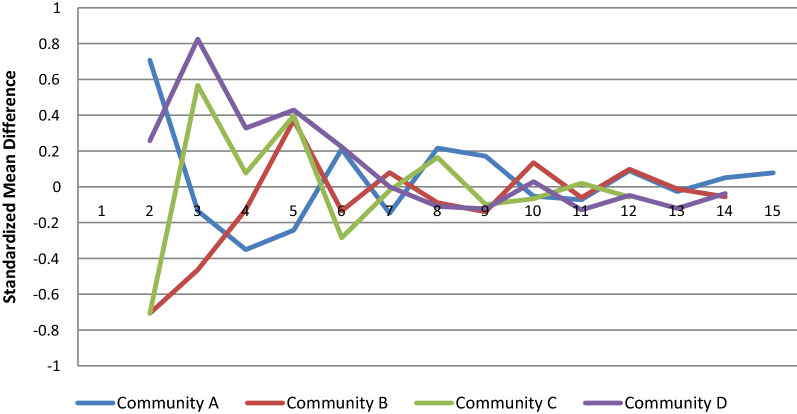
Standardized change in CR mean score over number of respondents (X axis)

Figure 2 displays the change in the CR score in the four communities when adding more interviews as standardized mean differences. After interviewing 10 key informants the change in the CR is smaller than ± 10% of the SD which translates into 0.08 CR scale points. Using a random order of respondents did not change the results suggesting that after interviewing 10 key informants little variation in the CR scores can be observed (Additional file 1: Figure S1 and S2).

Overall, a linear regression analysis confirmed a decline in newly identified issues with increasing number of interviews, although this association did not pass the threshold for statistical significance in the case of community efforts (Additional file 1: Table S1). Thus, there is a trend that with every additional interview the amount of new information gained from it becomes smaller. However, as can be seen in the (Additional file 1: Figure S3 to S5), even after completing 10 interviews in a community, respondents were still able to identify up to 6 new community efforts for PA promotion.

## Discussion

This study aimed to analyse whether increasing the number of key informants from 4–6 to 12–15 alters the results of the CRA. Overall, there were no large differences in the CR scores between the initial and the extended sample. Extending the sample did not change the mapping of the communities to a certain transtheoretical stage of change in the CR model. The CR scores showed little variation after having interviewed 10 key informants in a community, indicating that this may be a good number to strive for in CRA interviews. Our results are similar to a study by Guest et al. [10] who systematically examined the degree of data saturation in 60 in-depth interviews with women in two African countries. The authors started with analysing the first six interviews, then added a set of six more interviews, and so on. The process was repeated until all 60 interviews have been analysed. The authors found that data saturation occurred within the first 12 interviews with 92% of codes identified therein [10]. Two approaches on data saturation, code saturation (i.e., no additional issues raised, codebook stabilized) and meaning saturation (i.e., no further nuances or dimensions can be found), were examined in 25 in-depth interviews with HIV patients [9]. The authors found that code saturation was reached after nine interviews (91% of codes identified) and meaning saturation was achieved between 16 and 24 interviews. In addition, a study by Francis et al. [8] found that data saturation (i.e., minimum of 10 interviews, no new themes after analysing 3 further interviews) in theory-based interview studies was achieved after conducting 17 interviews. Focusing on studies applying the CR tool, Kostadinov et al. [6] included 40 articles published between 1999 and 2013 in a systematic review. The authors reported that the number of interviews per community varied between one and 33 (mean = 7.3, SD = 5.9, median = 6, six studies provided no information on the number of interviews per community), with 15% of studies using less than the recommended four interviews per community. This is in line with studies published since 2013 that followed the recommendations of the CR handbook and conducted an average of four to eight interviews per community (e.g., in [15–19]). However, Kesten et al. [20] used the concept of theoretical saturation (i.e., no new concepts are expected by conducting more interviews) to determine the necessary number of interviews for assessing CR for overweight and obesity prevention in pre-adolescent girls. Theoretical saturation was reached after conducting 33 interviews with key informants (e.g., parents, teachers). In addition, Pradeilles et al. [21] interviewed 12 key informants per community across two cities in Ghana, but did not give any reasons for selecting 12 interviewees per community. While it seems that conducting 10 interviews per community provides a fairly stable classification of a community in terms of their CR score, our results also indicate that there may still be unidentified issues regarding community efforts for PA promotion, contact and communication channel for informing or approaching the target group, or barriers to participation in PA promotion. A reason for this could be the nature of the PA efforts in the communities. There were many small scattered efforts and thus it appeared that the key informants did not mention all or were not aware of them. This indicates the context- and topic-relatedness of CRA, which needs to be kept in mind when planning similar studies.

## Limitations

Assuming stability of a mean if the change is less than 0.1 SD with every new respondent is an arbitrary choice. One could argue that small differences can lead to large changes if the sample is large and the differences go into the same direction. However, the results in our four communities did not indicate such a monotone pattern concerning the direction of change. Furthermore, there is a natural limit to the sample size of key informants. The number of potential key informants depends on the topic, the existing structures in the community, and the size of the community. For instance, in one of our selected communities it was not possible to identify more than 12 key informants who could provide information on community-based PA promotion in older adults. Our sampling strategy has the limitation that if key respondents recommend other potential key respondents (snowballing) the observations may not be independent from each other because the respondents may have shared views and attitude concerning the topic. Therefore we did not solely rely on snowballing but performed also internet searches to identify respondents. Another limitation is that in some cases key informants answered from their own point of view and may have had difficulties to respond on behalf of their community.

## Supplementary Information

**Additional file 1: Figure S1.** Mean CR score in 4 communities with random order of respondents. Number of participants are shown on the X axis. **Figure S2.** Standardized change in CR mean score over number of respondents (X axis) with random order of respondents. **Table S1.** Association between number of interviews and newly identified issues of CR (linear ordinary least square regression). **Figure S3.** Newly identified community efforts for the promotion of physical activity for older adults by number of key informants (X axis). **Figure S4.** Newly identified contact and information channels for the promotion of physical activity for older adults by number of key informants (X axis). **Figure S5.** Newly identified barriers to participation in physical activity for older adults by number of key informants (X axis).

## Data Availability

The data that support the findings of this study are available from the corresponding author on reasonable request.

## Abbreviations

CI: Confidence interval
CR: Community readiness
CRA: Community readiness assessment
PA: Physical activity
SD: Standard deviation
