# Supplementary material for: How many key informants are enough? Analysing the validity of the community readiness assessment
Source: BMC Res Notes. 2021 Mar 9;14:85. doi: 10.1186/s13104-021-05497-9 (PMC7941941; doi:10.1186/s13104-021-05497-9)
Supplement: Supplementary file 1 — Additional file 1: Figure S1. Mean CR score in 4 communities with random order of respondents. Number of participants are shown on the X axis. Figure S2. Standardized change in CR mean score over number of respondents (X axis) with random order of respondents. Table S1. Association between number of interviews and newly identified issues of CR (linear ordinary least square regression). Figure S3. Newly identified community efforts for the promotion of physical activity for older adults by number of key informants (X axis). Figure S4. Newly identified contact and information channels for the promotion of physical activity for older adults by number of key informants (X axis). Figure S5. Newly identified barriers to participation in physical activity for older adults by number of key informants (X axis). [file 13104_2021_5497_MOESM1_ESM.docx]

**Additional File**

**Figure S1. Mean CR score in 4 communities with random order of respondents. Number of participants are shown on the X axis**

**Figure S2. Standardized change in CR mean score over number of respondents (X axis) with random order of respondents**

**Table S1. Association between number of interviews and newly identified issues of CR (linear ordinary least square regression)**

|  | Model (1) | Model (2) | Model (3) |
| --- | --- | --- | --- |
|  | Community efforts | Communication channels | Barriers to participation |
|  |  |  |  |
| Number of respondents | -0.16 [-0.33, 0.02] | -0.16** [-0.26, -0.06] | -0.18*** [-0.28,-0.09] |
|  |  |  |  |
| Respondent group |  |  |  |
| Sports clubs/ facilities | Ref. | Ref. | Ref. |
| Civil services | 0.29 [-2.27, 2.84] | 1.14 [-0.34, 2.66] | 0.43 [-1.85, 1.00] |
| Public authorities | 0.88 [-0.70, 2.45] | 0.29 [-0.63, 1.20] | 0.14 [-1.02, 0.74] |
| Senior citizen advocacy groups | -0.69 [-2.81, 1.44] | -0.08 [-1.31, 1.15] | -0.91 [-2.09, 0.28] |
|  |  |  |  |
| Constant | 3.79*** [2.00, 5.59] | 2.96*** [1.92, 4.00] | 3.05*** [2.05, 4.05] |
|  |  |  |  |
| N | 55 | 55 | 55 |

Note: 95% confidence intervals in brackets, * p<0.05, ** p<0.01, *** p<0.001

**Figure S3. Newly identified community efforts for the promotion of physical activity for older adults by number of key informants (X axis)**

**Figure S4. Newly identified contact and information channels for the promotion of physical activity for older adults by number of key informants (X axis)**

**Figure S5. Newly identified barriers to participation in physical activity for older adults by number of key informants (X axis)**
